# Kombinierter Einfluss von psychologischen und biomechanischen Faktoren auf die muskulären Belastungen beim Fußballspielen: Ein neuer Ansatz zur Prävention von Muskelverletzungen?

**DOI:** 10.1007/s00132-023-04437-8

**Published:** 2023-10-02

**Authors:** Simon Auer, Simone Kubowitsch, Sebastian Dendorfer

**Affiliations:** 1 Labor für Biomechanik, Ostbayerische Technische Hochschule Regensburg, Seybothstraße 2, 93053 Regensburg, Deutschland; 2 Abteilung Wirtschaftspsychologie, Technische Hochschule Augsburg, Augsburg, Deutschland

**Keywords:** Biologische Modelle, Biomechanik, Leistungssportler, Bewegung, Resilienz, psychologische, Biological models, Biomechanics, Elite athletes, Movement, Resilience, psychological

## Abstract

Beim Zusammenwirken von mentaler Beanspruchung und muskuloskelettaler Belastung steigt das Risiko für Verletzungen durch veränderte Körperkinematik und erhöhte Muskelspannung. Diese Veränderungen können mit muskuloskelettalen Modellen festgestellt werden, wobei zusätzlich die mentale Belastung und Beanspruchung auf emotionaler, kognitiver und verhaltensbezogener Ebene analysiert werden muss. Um diese Kinematik- und Belastungsänderungen unter Stress zu untersuchen, wurden Leistungssportler:innen bei hochdynamischen Bewegungen mentalem Stress ausgesetzt und mittels muskuloskelettaler Modelle die biomechanische Belastung analysiert. Dabei zeigte sich, dass es unter mentaler Beanspruchung, unabhängig vom subjektiven Empfinden, zu einer starken Änderung der Muskelkräfte kommen kann. Entsprechend sollten Leistungssportler:innen Screenings zur Beurteilung der individuellen Bewegungsmuster durchlaufen und die allgemeine Stressresilienz gefördert werden.

## Abstract (en)

When mental stress and musculoskeletal loading interact, the risk for injury increases due to altered body kinematics and increased muscle tension. These changes can be detected with musculoskeletal models, and mental loading and stress must be analyzed at emotional, cognitive, and behavioral levels. To investigate these kinematic and loading changes under stress, competitive athletes were subjected to mental stress during highly dynamic movements, and musculoskeletal models were used to analyze the biomechanical loading. It was shown that under mental stress, independent of the subjective perception, a strong change in muscle forces can occur. Accordingly, competitive athletes should undergo screenings to assess individual movement patterns and promote general stress resilience.

Für Fußballer:innen stellen muskuläre Verletzungen der unteren Extremitäten ein großes Problem dar. Ein Beispiel hierfür liefert die Nationalmannschaftsstürmerin Alexandra Popp, die aufgrund muskulärer Probleme das EM-Finale 2022 in Wembley kurzfristig verpasste. Oftmals stehen gerade hohe Anspannungssituationen in zeitlichem Zusammenhang mit Verletzungen, der Einfluss der psychischen Beanspruchung auf die biomechanischen Belastungen wird jedoch meist nur wenig beachtet.

## Einleitung

Verletzungen der Oberschenkelmuskulatur machen mit ca. 30 % einen Großteil der Verletzungen von Profifußballer:innen aus [5]. Sie sind besonders problematisch, da sie zu langen Ausfallzeiten und hohen Behandlungskosten führen können. Generell ist die Verletzungshäufigkeit während des Spiels deutlich höher als während des Trainings (35 Verletzungen pro 1000 h vs. 4 Verletzungen pro 1000 h) [14]. Die Ursachen sind jedoch vielfältig und werden von äußeren und inneren Faktoren beeinflusst. Faktoren wie (Schutz‑)Ausrüstung, Umgebung, Wetter und Situation wirken sich ebenso auf die Sportler:innen aus, wie die individuelle Anatomie und Physiologie, der Fitnesszustand, die persönlichen Fähigkeiten und eventuelle Vorverletzungen. Zu diesen Einflüssen kommen weitere Aspekte wie Dauerbelastung, auftretende Kinematik oder Krafteinwirkung hinzu [7, 15]. All diese Faktoren beeinflussen das Auftreten, die Art und den Schweregrad von Verletzungen.

Studienergebnisse weisen darauf hin, dass auch psychologische Faktoren mit dem Risiko von Sportverletzungen in Verbindung stehen. Als Indikatoren wurden Persönlichkeitsmerkmale wie Ängstlichkeit und eine damit verbundene verminderte Fähigkeit, mit belastenden Situationen in Form von negativen Lebensereignissen oder Alltagsstress umzugehen, ermittelt. Dies wurde in Querschnitts- und prospektiven Studien belegt [1, 9]. Neben dieser individuellen Ebene lassen sich Risikofaktoren zusätzlich im interpersonellen Bereich identifizieren. Dies zeigt sich etwa in Zusammenhängen zwischen der Beziehung von Trainer und Athlet und einem erhöhten Verletzungsrisiko [19]. Auch Vorverletzungen stellen einen Stressfaktor dar, der zu erhöhter Ängstlichkeit und damit zu einem Teufelskreis führen kann [10]. Das Verletzungsrisiko steigt auch zusätzlich mit der Dauer der Belastung. So führt beispielsweise eine gesteigerte Anspannung über 3–4 Wochen zu einem deutlich erhöhten Verletzungsrisiko [15].

Zur Beurteilung der Verletzungsgefährdung müssen somit multiple Faktoren berücksichtigt werden, die direkt oder indirekt auf die Muskulatur einwirken. In diesem Zusammenhang möchten wir die kombinierten Einflüsse von biomechanischen und psychologischen Belastungen diskutieren.

## Kaskade der Belastungen

**Figure 1:**
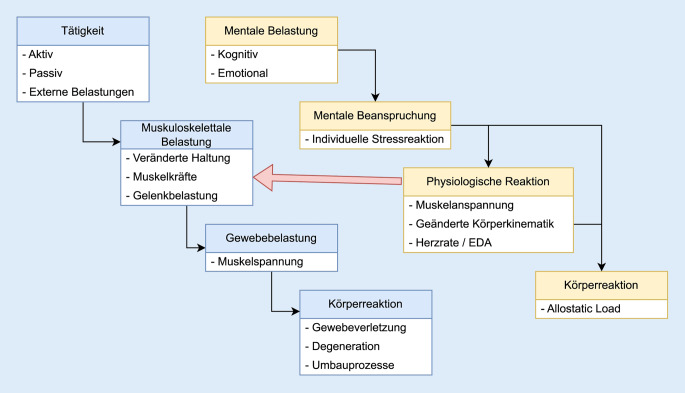

Die Einwirkung von biomechanischen und auch mentalen Belastungen kann vereinfacht in einer Kaskadenstruktur zusammengefasst werden (Abb. 1). Bei den biomechanischen Belastungen führen Tätigkeiten wie Sprints in der ersten Stufe zu muskuloskelettalen Belastungen in Form von z. B. Muskel- und Gelenkkräften oder Bänderdehnungen. Die integralen Belastungen von Strukturen können auch auf einer weiteren Diskretisierungsebene als Gewebebelastungen wie z. B. Muskelspannung oder Gelenkpressung dargestellt werden. Aus strukturmechanischer Sicht wird diese Ebene in der Regel zur weiteren Beschreibung von Veränderungen der Gewebestruktur, wie der Entstehung von Verletzungen, oder der Initiierung von Umbauprozessen oder Degenerationen verwendet.

Analog dazu kann in einer ersten Stufe die Einwirkung von mentalen Belastungen, etwa kognitiver oder emotionaler Art, in Abhängigkeit von moderierenden Faktoren, wie individuellen Bewertungsprozessen, zu mentalen Beanspruchungen führen, aus denen eine psychische Reaktion resultieren kann. Typische messbare körperliche Reaktionen auf eine höhere mentale Beanspruchung sind eine veränderte Herzrate und Hautleitwiderstand, eine Erhöhung des Muskeltonus und eine geänderte Körperkinematik. Hier ergibt sich eine offensichtliche Querverbindung zwischen mentalen und biomechanischen Belastungen. Die physiologischen Reaktionen auf die mentalen Belastungen beeinflussen die biomechanischen Belastungen direkt auf muskulärer Ebene, durch eine Erhöhung der Vorspannung und bedingt durch die geänderte Körperkinematik.

## Belastungsanalyse mit muskuloskelettalen Modellen

**Figure 2:**
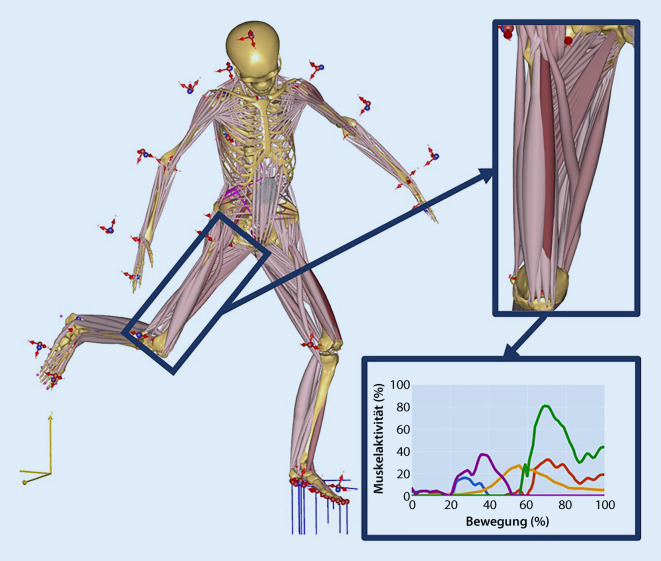

**Figure 3:**
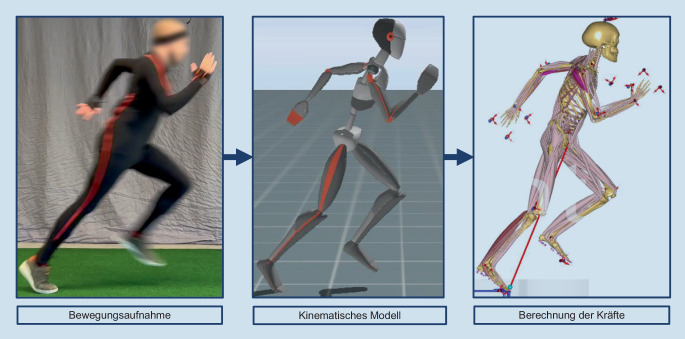

Zur Bestimmung biomechanischer Belastungen wurden in den letzten Jahren verstärkt validierte muskuloskelettale Simulationsmodelle verwendet. Diese wurden bisher z. B. erfolgreich für orthopädische oder ergonomische Studien eingesetzt [3, 18, 22]. Muskuloskelettale Modelle sind numerische Abbildungen des Menschen (Abb. 2), mit denen interne (z. B. Muskel- oder Gelenkreaktionskräfte) oder auch externe Kräfte (z. B. Bodenreaktions- oder Kontaktkräfte) berechnet werden können. Grundlage für die Berechnungen sind meist Bewegungsaufnahmen mit Motion-Capture-Systemen (Abb. 3). Der Goldstandard sind hier markerbasierte Systeme, bei denen reflektierende Marker an anatomischen Landmarken auf die Haut geklebt und von Infrarotkameras erfasst werden. Diese Technik ermöglicht ein genaues Tracking der menschlichen Bewegung und folglich auch eine genaue Abbildung der Bewegung im muskuloskelettalen Modell. Die Genauigkeit dieser Bewegungsaufnahmen und die Validität der muskuloskelettalen Modelle wurde in den letzten beiden Jahrzehnten vielfach untersucht und bestätigt. In den letzten Jahren fanden allerdings auch mehr und mehr neue Messmethoden Verwendung, welche die Bewegungsaufnahme vereinfachen, beispielweise basierend auf Trägheitssensoren, weiteren Wearables oder Videoaufnahmen.

### Bestimmung von mentaler Belastung und Beanspruchung

Wie bereits erwähnt, beeinflussen auf psychologischer Ebene nicht nur individuelle Merkmale, sondern auch auf eine Person einwirkende Stressoren sowie deren Bewältigungsstrategien das Verletzungsrisiko. Dies lässt sich im Sinne eines transaktionalen Stressverständnisses einordnen [6, 12]. Nach dieser Modellvorstellung hängt eine Stressantwort maßgeblich davon ab, wie ein Individuum die einwirkenden Stressoren bewertet. Insbesondere wenn die verfügbaren Ressourcen zur Bewältigung des Stressors subjektiv als nicht ausreichend eingeschätzt werden, tritt eine Stressreaktion ein.

Konzeptuell ist so zwischen Belastung und Beanspruchung zu differenzieren. Belastung bezieht sich auf die objektiv von außen auf eine Person wirkenden Stressoren, Beanspruchung hingegen auf die individuelle Stressreaktion einer Person. Für Monitoring der psychischen Belastung können Fragebögen und Checklisten zum Vorliegen von Stressoren im sportbezogenen und persönlichen Bereich verwendet werden [17].

Eine Messung der Beanspruchung kann auf mehreren Ebenen erfolgen. Hier sind körperliche, emotionale, kognitive und verhaltensbezogene Reaktionen zu unterscheiden. Besonders bedeutsame Parameter sind in diesem Kontext Messungen der Veränderung des Muskeltonus über Oberflächenelektromyografie und der Körperkinematik über Bewegungsanalysesysteme. Auf diese Weise lässt sich die Interaktion von biomechanischen und psychologischen Variablen abbilden. Um auch kumulative Effekte durch längerfristig einwirkende Stressoren zu erfassen, können entsprechend des Konzeptes der Allostatic Load von McEwen [16] auf neuroendokrinologische, kardiovaskuläre, metabolische und immunologische Parameter zurückgegriffen werden.

Da individuelle Merkmale den Zusammenhang zwischen Belastungen und erlebter Beanspruchung moderieren, sollten in diesem Kontext sowohl die Stressreaktion verschärfende Faktoren wie Ängstlichkeit als auch abmildernde Faktoren wie soziale Unterstützung und Selbstwirksamkeit berücksichtigt werden.

### Starke Änderung der Muskelkräfte unter Stress

Während der Einfluss von mentaler Belastung auf den Muskeltonus in einer Reihe von Studien für verschiedenste Szenarien beschrieben wurde, gibt es bisher kaum Studien, die den Zusammenhang von muskulärer Belastung und mentalem Stress fußballspezifisch untersucht haben. Ebenso wurde die Auswirkung von mentalem Stress auf die Biomechanik bisher kaum mithilfe von muskuloskelettalen Modellen untersucht, die, wie oben beschrieben, eine detaillierte Betrachtung der wirkenden biomechanischen Lasten zulassen. In einer kürzlich durchgeführten Studie der Autoren wurden diese Zusammenhänge untersucht. Analysiert wurden zwölf Jugendleistungsfußballer, die während hochdynamischer Läufe mentalem Stress ausgesetzt wurden [2]. Die Fußballer wurden während ihrer Läufe in einem Speedcourt (Globalspeed GmbH, Hemsbach, Deutschland), mit einem kognitiven Stressor belastet, bei dem sie in einer Reihe von „d“ und „p“ mit variierender Anzahl von Strichen über und unter dem Buchstaben die „d“ mit exakt 2 Strichen erkennen mussten [4]. So wurde eine aufmerksamkeitsfordernde und kognitiv herausfordernde Stresssituation, ähnlich dem Antizipieren einer dynamischen Spielsituation, geschaffen. Die Körperkinematik wurde mit 12 Kameras eines markerbasierten Motion-Capture-Systems (Vicon Motion Systems, Oxford, Großbritannien) aufgezeichnet und die Muskelkräfte mit und ohne Stressor mit muskuloskelettalen Modellen (AnyBody Modeling System, Aalborg, Dänemark) berechnet. Ebenfalls wurde mithilfe von Fragebögen die mentale und körperliche Beanspruchung erhoben. Es zeigte sich, dass die Sportler eine höhere subjektive Beanspruchung unter Stress angaben (+27,5 %) und gleichzeitig signifikant langsamer liefen (−12 %). Die biomechanischen Analysen von insgesamt 408 Datensätzen ergaben ein sehr uneindeutiges Bild. Während es bei manchen Probanden trotz angegebener subjektiver Beanspruchungserhöhung zu keiner Änderung der Muskelkräfte im Vergleich zum ungestressten Lauf gab, konnte bei den meisten Probanden eine Belastungsänderung in Form von deutlich erhöhter oder verringerter aufgebrachter Muskelkraft festgestellt werden. Insbesondere die erhöhten Muskelkräfte sind im Kontext der geringeren Laufgeschwindigkeiten unter Stress interessant. Beispielsweise konnten bei zwei Probanden (Beispiele A und B, Tab. 1) keine signifikant veränderten Muskelkräfte unter Stress festgestellt werden, obwohl diese die mentale Beanspruchung um bis zu 60 % höher einschätzten. Im Gegensatz dazu stehen Probanden, bei denen das subjektive Beanspruchungsempfinden mit der Veränderung der Muskelbelastung korreliert (Beispiel C, Tab. 1) und sich die Muskelkräfte unter Stress um bis zu 146 %BW änderten. Auch ein geringeres Beanspruchungsempfinden ist kein sicherer Hinweis auf adäquate Strategien zum Umgang mit Stress, da es auch bei Probanden mit geringerem Belastungsempfinden (−20 %) zu einer Belastungserhöhung von bis zu 65 %BW kam. Für die Veränderung der Muskelkräfte zeigte sich so kein Zusammenhang zum subjektiven Beanspruchungsempfinden, sehr wohl jedoch zur Fehlerrate der kognitiven Aufgabe. Dies deutet auf unterschiedliche Priorisierungsprozesse der Sportler hinsichtlich der motorischen und kognitiven Aufgabe hin.

**Table 1:** 

Proband	Maximale (relative) Änderung der Muskelkraft unter Stress	(Relative) Änderung der für den Lauf benötigten Zeit	Empfundene mentale Belastung
A	+23 %BW (18 %)	+4 s (11 %)	+50 %
B	−18 %BW (9 %)	+7 s (20 %)	+60 %
C	−146 %BW (50 %)	±0 s	+20 %
D	+65 %BW (37 %)	+4 s (10 %)	−20 %

*%BW* Prozent Körpergewicht (body weight)

Insgesamt ist anzumerken, dass eine mögliche Erhöhung des Muskeltonus, die nicht auf einer geänderten Kinematik beruht, hier noch nicht berücksichtigt ist. Dies konnte in einer weiteren Studie bei einer einfachen standardisierten Flexions-Extensions-Bewegung des Oberkörpers unter dem Einfluss eines kurzfristig wirkenden kognitiven Stressors gezeigt werden [11, 20]. Bemerkenswert ist auch hier, dass sich nicht ein einziges, sondern zumindest zwei sich unterscheidende Reaktionsmuster unter Stress feststellen ließen. Diese beiden Gruppen unterschieden sich hinsichtlich der Muskelaktivierung und auch der Bewegungsgeschwindigkeit signifikant.

Stressbedingte Änderungen in der Kinematik konnten grundlegend auch in anderen Studien beschrieben werden. So stellten z. B. Higuchi et al. [8] veränderte Bewegungsstrategien unter Stress fest. Lohse und Sherwood [13] schlugen vor, dass eine innere Fokussierung der Aufmerksamkeit die effiziente motorische Kontrolle stört. Vor diesem Hintergrund können die Auf- und Abwärtsschwankungen der Spitzenmuskelkraft durch ein verändertes Bewegungsverhalten und eine unphysiologische Muskelaktivierung in Form einer gestörten effizienten motorischen Kontrolle unter mentalem Stress hervorgerufen werden [21].

Die Reaktion auf einen zusätzlichen mentalen Stressor ist sehr individuell

Aufgrund der eher geringen Probandenzahl lassen sich zwar keine allgemeingültigen Schlussfolgerungen auf die Muskelbelastung unter Stress ziehen, jedoch kann festgestellt werden, dass die Reaktion auf einen zusätzlichen mentalen Stressor sehr individuell und unabhängig vom subjektiven Empfinden ist. Auffällig ist die hohe individuelle Varianz der körperlichen Antwort auf die Stressbelastung. Hier sind dringend weitere Studien notwendig, um die Einflüsse besser einordnen zu können.

## Präventionsmaßnahmen

Neben vielfältigen weiteren Einflüssen auf das potenzielle Verletzungsrisiko ergeben sich für die kombinierte biomechanische und mentale Beanspruchung mehrere mögliche Ansatzpunkte. Auf psychologischer Ebene lassen sich in einem ersten Schritt die einwirkenden Stressoren und individuellen Risiko- aber auch Schutzfaktoren systematisch analysieren und daraus erste effektive Präventionsmaßnahmen ableiten. Ein Beispiel hierfür ist die vorausschauende Planung der Einsatzzeiten im weiteren Saisonverlauf gemeinsam mit dem Trainerteam im Rahmen des Belastungsmanagements.

Zusätzlich gilt es, potenziell verletzungsträchtige Bewegungen in auftretenden Anspannungssituationen zu vermeiden sowie adäquate mentale Coping-Strategien auf einwirkende Belastungen zu erlernen und damit die allgemeine Stressresilienz zu erhöhen. Diese allgemeinen Präventionsstrategien sind sinnvoll, in diesem speziellen Setting jedoch wahrscheinlich nicht ausreichend und zu unspezifisch.

Die interindividuell stark variierenden muskulären, kinematischen und psychischen Reaktionen auf Stressoren erfordern Kenntnisse über das individuelle Verhalten während Anspannungssituationen. Deshalb sind Screenings in wettkampfnahen Szenarien zur Beurteilung der individuellen Reaktionsmuster notwendig. Hier kommt der Analyse der individuellen Priorisierungsstrategien eine Hauptrolle zu. Deshalb ist eine Kombination aus biomechanischen und psychologischen Tests nötig, um ein umfassendes Bild der individuellen Antwort von Sportlern zu erhalten. Der Umgang mit diesen Stressreaktionen kann in einem weiteren Schritt über eine positive Beeinflussung von kinematischen und physiologischen Veränderungen trainiert werden.

Technologisch werden diese Screenings von neuen Entwicklungen in der Sensorik und Messtechnik gestützt. Beispielsweise können durch markerlose Bewegungsmesssysteme auch außerhalb eines Laborsettings detaillierte kinematische Daten erhoben werden, welche wie beschrieben als Grundlage für weitergehende Analysen und Interventionen benötigt werden.

## Fazit für die Praxis

- Mentaler Stress kann die biomechanischen Belastungen signifikant erhöhen.
- Mentaler Stress kann sowohl die Bewegungsmuster wie auch die Muskelanspannung ändern.
- Die Stärke der Auswirkungen von mentalem Stress variiert individuell sehr stark.
- Screening-Maßnahmen für die kombinierte psychologisch-biomechanische Reaktion werden empfohlen.

## Abkürzungen

%BW: Prozent Körpergewicht (body weight)
EDA: Hautleitfähigkeit
EM: Europameisterschaft
